# Overt speech decoding from cortical activity: a comparison of different linear methods

**DOI:** 10.3389/fnhum.2023.1124065

**Published:** 2023-06-23

**Authors:** Gaël Le Godais, Philémon Roussel, Florent Bocquelet, Marc Aubert, Philippe Kahane, Stéphan Chabardès, Blaise Yvert

**Affiliations:** ^1^Univ. Grenoble Alpes, INSERM, U1216, Grenoble Institut Neurosciences, Grenoble, France; ^2^CHU Grenoble Alpes, Department of Neurology, Grenoble, France; ^3^Univ. Grenoble Alpes, CHU Grenoble Alpes, Clinatec, Grenoble, France

**Keywords:** decoding, ECoG, brain-computer interface, linear methods, speech prostheses, intracranial recordings, articulatory synthesis

## Abstract

**Introduction:**

Speech BCIs aim at reconstructing speech in real time from ongoing cortical activity. Ideal BCIs would need to reconstruct speech audio signal frame by frame on a millisecond-timescale. Such approaches require fast computation. In this respect, linear decoder are good candidates and have been widely used in motor BCIs. Yet, they have been very seldomly studied for speech reconstruction, and never for reconstruction of articulatory movements from intracranial activity. Here, we compared vanilla linear regression, ridge-regularized linear regressions, and partial least squares regressions for offline decoding of overt speech from cortical activity.

**Methods:**

Two decoding paradigms were investigated: (1) direct decoding of acoustic vocoder features of speech, and (2) indirect decoding of vocoder features through an intermediate articulatory representation chained with a real-time-compatible DNN-based articulatory-to-acoustic synthesizer. Participant's articulatory trajectories were estimated from an electromagnetic-articulography dataset using dynamic time warping. The accuracy of the decoders was evaluated by computing correlations between original and reconstructed features.

**Results:**

We found that similar performance was achieved by all linear methods well above chance levels, albeit without reaching intelligibility. Direct and indirect methods achieved comparable performance, with an advantage for direct decoding.

**Discussion:**

Future work will address the development of an improved neural speech decoder compatible with fast frame-by-frame speech reconstruction from ongoing activity at a millisecond timescale.

## 1. Introduction

Recent advances of intracranial brain-computer interfaces (BCIs) have opened the possibility for paralyzed people to communicate through devices such as cursors to spell on a virtual keyboard (Serruya et al., 2002; Hochberg et al., 2006; Jarosiewicz et al., 2015; Pandarinath et al., 2018) or by reconstructing imagined handwritten letters (Willett et al., 2021). The increased efficiency of these systems allowed to reach performance close to regular typing on a smartphone. Yet, such BCIs do not use speech-related cortical activity to perform a communication task, and thus remain less intuitive to control than natural speech. Moreover they largely require motor resources unrelated to speech, preventing from simultaneously using a motor BCI. Speech BCIs effectively controlled by speech activity have been proposed to either classify discrete representations of speech such as letters (Metzger et al., 2022) and words (Moses et al., 2021), or to decode continuous features of speech such as formants (Guenther et al., 2009) and detailed acoustic representations of whole speech (Pasley et al., 2012; Martin et al., 2014; Angrick et al., 2021).

Considering the clinical difficulty to implant electrodes for the purpose of a dedicated speech BCI, many studies have used data from patients implanted for other clinical purposes. Offline decoding of discrete speech units has been explored by classifying phonemes (Mugler et al., 2014; Herff et al., 2015), vowels (Tankus et al., 2012; Ibayashi et al., 2018), words (Kellis et al., 2010), or entire sentences (Moses et al., 2019). Other studies focused on offline decoding of continuous representations of speech which are language-agnostic, in contrast with discrete representations. Spectrograms of perceived words and sentences from a limited set have been reconstructed from the auditory cortex by linear regression (Pasley et al., 2012), and spectrograms of produced speech have been decoded from electrocorticography (ECoG) recordings of temporal areas (Herff et al., 2016). Other studies improved over spectrograms by decoding vocoder features of speech including voicing, F0, aperiodicity and spectral envelope (Akbari et al., 2019). Finally, decoding a compact intermediate articulatory representation of speech chained with an articulatory-to-acoustic synthesizer could allow to control a speech BCI with fewer parameters (Bocquelet et al., 2016a), as articulatory trajectories are also a language-agnostic representation of speech that is encoded in the cortex (Bouchard et al., 2013; Chartier et al., 2018; Conant et al., 2018). Such an approach has been found to improve performance over the direct decoding of vocoder features of speech, achieving high-quality reconstruction of speech sentences (Anumanchipalli et al., 2019). The method was however not compatible with real-time reconstruction of continuous speech as sentences were processed as whole.

A natural speech BCI would allow intuitive production of arbitrary speech in real-time so that a conversation could be carried smoothly. To control a speech synthesizer in real-time, the BCI would require a neural decoder that could be integrated in a closed-loop process computable within milliseconds (Bocquelet et al., 2016c). Having the advantage of fast computation, linear decoder are good candidates and have already been widely used for motor BCIs (Hochberg et al., 2006, 2012; Collinger et al., 2013; Wodlinger et al., 2015). However, they have been tested only very seldom for continuous decoding of produced speech from ongoing brain activity (Martin et al., 2014). In particular, their capacity to decode articulatory movements from intracranial activity has not yet been explored and different linear methods have not been compared. In this context, we evaluate here different linear decoders for continuous speech decoding from Electrocorticography (ECoG) activity. We investigate direct decoding of acoustic vocoder features of speech as well as indirect decoding of vocoder features through an intermediate articulatory representation chained with a real-time-compatible Deep Neural Network-based (DNN) articulatory-to-acoustic synthesizer (Bocquelet et al., 2014, 2016c; Anumanchipalli et al., 2019).

## 2. Methods

### 2.1. Data

This work required synchronized recordings of audio, articulatory trajectories and neural activity of speech. The chosen methodologies were Electromagnetic Articulography (EMA) for recording articulatory trajectories, and Electrocorticography (ECoG) for brain activity. As simultaneous EMA and ECoG recordings were not allowed by the regulatory constraints of the experiment protocol, separate EMA and ECoG datasets were considered.

#### 2.1.1. EMA data: BY2014

BY2014 (Bocquelet et al., 2016b) is a large articulatory-acoustic corpus containing the recording of vocal tract movements and simultaneous audio signals in one French male speaker reading 676 short sentences including isolated vowels and VCVs (vowel-consonant-vowel sequences like “*apa”*, “*iti”*,...). EMA was recorded with 9 3-dimensional sensors at 100 Hz positioned on lips corners, upper and lower lips, tongue tip, back and dorsum, soft palate, and jaw (actually front teeth). Head movements were removed from the recordings so that articulatory trajectories are describing movements relatively to the head. The corpus therefore consists of 27 articulatory features and the synchronized audio recording.

#### 2.1.2. ECoG data: P5

This study is part of the Brainspeak clinical trial (NCT02783391) approved by the French regulatory agency (DMDPT-TECH/MM/2015-A00108-41) and the local ethical committee (CPP-15-CHUG-12). It is based on electrophysiological recordings obtained in P5, a female participant implanted for 7 days at Grenoble University Hospital as part of a presurgical evaluation of her intractable epilepsy. P5 gave her informed consent to participate in the study.

##### 2.1.2.1. Recordings

Brain activity was recorded in the participant's room at the hospital. P5 was implanted with a 72-electrode array (PMT Corp., USA) covering a large portion of her left hemisphere as well as a 4-electrode strip (PMT Corp., USA) over the left ventral temporal lobe. One electrode of the strip was used as the reference and one as the ground. An additional 96-electrode microelectrode array was also implanted in the participant's cortex but was not used in this work. The audio and brain signals were amplified and synchronously recorded at 30 kHz.

##### 2.1.2.2. Task

P5 was asked to read aloud a set of short French sentences from BY2014 dataset (see Section 2.1.1). During the 4-day experiment, P5 participated to both closed loop and open loop tasks. During **open loop** experiments, P5 read sequences of vowels and short sentences from BY2014 without any audio feedback. Depending on the recording sessions, P5 produced each sentence following multiple speaking conditions: first reading, then repeating the same sentences, and lastly covertly repeating it again before saying “*ok”* when done. Both *read* and *repeat* conditions required to speak out loud, but the written sentence was only displayed on the screen during *read* condition. For the *covert* condition, P5 was asked to imagine repeating the sentence once more, without actually producing speech or moving the articulators, and without seeing it on screen.

For this work, only open loop recordings of the three first days of experiments were used, in overt conditions (*read* and *repeat*). During day 1, P5 read and repeated 97 sentences including 4 repetitions of 4 vowel sequences (“*a, i, ou”*; “*u, é, è”*; “*e, o, an”*; and “*on, in”*). During day 2, P5 read 141 sentences, including 6 repetitions of 4 vowel sequences. During day 3, P5 read and repeated 153 sentences, including 7 repetitions of 4 vowels sequences. This amounts to a total of 391 read sentences and 250 repeated sentences, thus 641 sentences in total.

##### 2.1.2.3. Annotation

All sentences were manually inspected one by one to annotate the condition, transcription, phonetic transcription and if necessary to discard failed attempts or trials with noisy backgrounds. Sentences were automatically cut using a speech envelope detection (see Section 2.2.2) so that only 500 ms of silence remained before and after speech, although some manual adjusting was necessary. The annotated speech conditions were *read, repeat, covert*, and *rest* that labeled resting intervals in between trials.

### 2.2. Neural data processing

#### 2.2.1. Preprocessing

Artifacts such as line noise were removed from neural signals using common median reference. At each time step, the median value of all channels was computed. The resulting signal was subtracted from all channels to remove noise that was shared between all electrodes, such as line noise or electromagnetic interferences. Removing the median signal was found to be more robust to outliers than removing the average signal.

Some ECoG recordings have been found to be contaminated by acoustic signals (Roussel et al., 2020). P5 dataset was investigated for acoustic contamination using a Matlab package available on Zenodo (Roussel et al., 2021). It was found that none of the recording sessions of P5 dataset used in this work contained significant acoustic contamination. Moreover, given the high-pitched voice of P5, any contamination would only affect frequencies above 200 Hz, which is higher than the neural features that were used for speech decoding (see Section 2.2.3).

#### 2.2.2. Automatic speech detection

The audio envelope was extracted from the audio recordings using the Hilbert transform. A smoothed envelope was then computed by running a moving average with a 100 ms window on the audio envelope. A threshold for speech detection was set at 10% of the maximum smoothed envelope value. Any segment of the smoothed envelope crossing the threshold for at least 50 ms was considered to contain a vocalization. Finally, speech segments that were under 100 ms from each other were merged into one.

#### 2.2.3. Neural features

**Spectrograms** were computed from neural signals using a Fast Fourier Transform with a moving hamming window of 200 ms, a 10 ms frame shift, and padding by symmetrizing the signal. The power spectral density of each frequency bands was averaged over 10 Hz bands from 0 to 200 Hz, resulting in 20 spectral features sampled at 100 Hz. Additionally, the raw signal filtered between 0.5 and 5 Hz was used as an additional feature for each electrode. A total of 21 neural features were thus computed for each electrode signal of the ECoG dataset.

#### 2.2.4. Frontal and temporal electrodes

In a dedicated analysis, P5 neural features were split into frontal and temporal categories. All features from electrodes placed above the lateral sulcus were considered as frontal, while the remaining features were considered as temporal (representation in **Figure 5A**). With these categories, 28 electrodes were categorized as frontal, and 44 as temporal. The frontal electrodes covered the areas responsible for speech motor control, while the temporal electrodes covered the auditory regions.

#### 2.2.5. Context and delays

Actual speech production of the sound wave and its underlying cognitive processes are typically not simultaneous. Indeed, the motor control of articulators requires planning and therefore happens before sound production, while the processing of auditory and somatosensory feedback happens after sound production. In order to take into account these cognitive processes for speech decoding, two parameters were considered for neural decoding: 1. the **time delay** between the center of the time context window of neural features and the decoded time of acoustic/articulatory features of speech, and 2. the **time context** that consisted in concatenating multiple consecutive frames of neural features to decode one frame of acoustic/articulatory features of speech. By convention, a time context of 110 ms corresponded to concatenating frames of neural features **x**(*t* − 50*ms*)…**x**(*t* + 50*ms*), and a time delay τ corresponded to synchronizing the neural features **x**(*t*) with the acoustic features **y**(*t* + τ).

### 2.3. Acoustic data processing

#### 2.3.1. Preprocessing

Any DC offset was removed from audio sentences by subtracting their mean value from the signal. Resulting signals were then peak-normalized and their average volume was set to −20 dB using automatic gain control in Matlab. Lastly, P5 sentences were resampled at 22,050 Hz to mach BY2014's sampling rate using Matlab's *resample* function with the default anti-aliasing lowpass filter.

#### 2.3.2. Source-filter representation

A Mel cepstral and F0 analysis of speech was computed from audio recordings using SPTK (Imai, 2003). This source filter representation was motivated by the possibility for real-time synthesis of speech using a Mel Log Spectral Approximation (MLSA) filter (Bocquelet et al., 2016c), and was also implemented in SPTK.

##### 2.3.2.1. Mel cepstrum

Mel cepstrums of order 24 were extracted from audio recordings using SPTK. The signal analysis was performed with Blackman windows of 400 samples in input and 1,024 in output with quadratic normalization, a frame shift of 220 samples and a frame length of 1,024. The ε parameter was set to 10^−4^ to avoid errors in the periodogram computations. The all-pass constant α was set to 0.455 to accurately estimate the Mel scale for a 22,050 Hz sampling rate.

Due to the 220 sample frame shift, the resulting 25 Mel cepstral coefficients were sampled at ~100.23 Hz. Each sentence was then resampled to 100 Hz in order to accurately match articulatory and neural features sampling rate. The resampling was performed by shape-preserving piecewise cubic interpolation of the signal with the “*pchip”* parameter of Matlab's *interp1* function.

##### 2.3.2.2. F0

The F0 was extracted from P5 dataset using the SWIPE' algorithm from SPTK. The parameters for signal windowing were the exact same parameters used to extract Mel cepstral coefficients described in Section 2.3.2.1. After visual inspection of the dataset's spectrograms, the F0 search algorithm was constrained to 80–300 Hz. The extracted F0 signals are set to the fundamental frequency when the signal is voiced and 0 when it is unvoiced.

Like Mel cepstral coefficients, the extracted F0 was resampled to 100 Hz to match articulatory and neural features. Due to the discontinuities in the F0 that had to be preserved, resampling was done with a nearest neighbor interpolation. F0 misdetections were filtered out by removing any F0 segment shorter than 50 ms, after visual inspection of the data.

#### 2.3.3. Synthesis

Speech audio was synthesized from Mel cepstrum and F0 using SPTK's MLSA filter. The MLSA filter was excited by either a white noise source for voiceless signals, or an impulse train with a period changing according to the F0 for voiced signals. SPTK refers to this period as *pitch*, and requires it as the parameter controlling the generation of the excitation signal. A period of 0 defines by convention that no F0 is detected, and that the excitation signal should be white noise. Given a frame rate *f*_*s*_ (22,050 Hz here), pitch was therefore reconstructed from F0 with the formula:


(1) $$pitch=\{\begin{array}{ll} \frac{f_{s}}{f_{0}}\; & \text{if}\;f_{0}\neq 0\\ 0\; & \text{if}\;f_{0}=0 \end{array}$$

Processing of the excitation signal and MLSA synthesis used the same α parameter and frame period as for the Mel cepstral analysis presented in Section 2.3.2.1. Output waveforms were peak-normalized and limited to avoid clipping, and loudness was set to −20 dB using automatic gain control.

### 2.4. Articulatory data processing

#### 2.4.1. Articulatory data

Articulatory trajectories recorded by 3D EMA contain a lot of redundant information, as most of the trajectories can be characterized in the midsagittal plane. Thus, each sensor was projected on the midsagittal plane of the speaker using Principal Component Analysis (PCA) and keeping only the first two components. As the 2 lips corners mostly move along the latero-medial axis, they were removed from the 9 original sensors of BY2014. The resulting 14 articulatory features tracked the 2D trajectories of the upper and lower lips; tongue tip, back and dorsum; velum; and jaw.

In order to decode articulatory trajectories from P5's neural activity, those articulatory trajectories had to be inferred from BY2014 using the method described in Section 2.4.2.

#### 2.4.2. Estimation of articulatory trajectories

**Figure 1:**
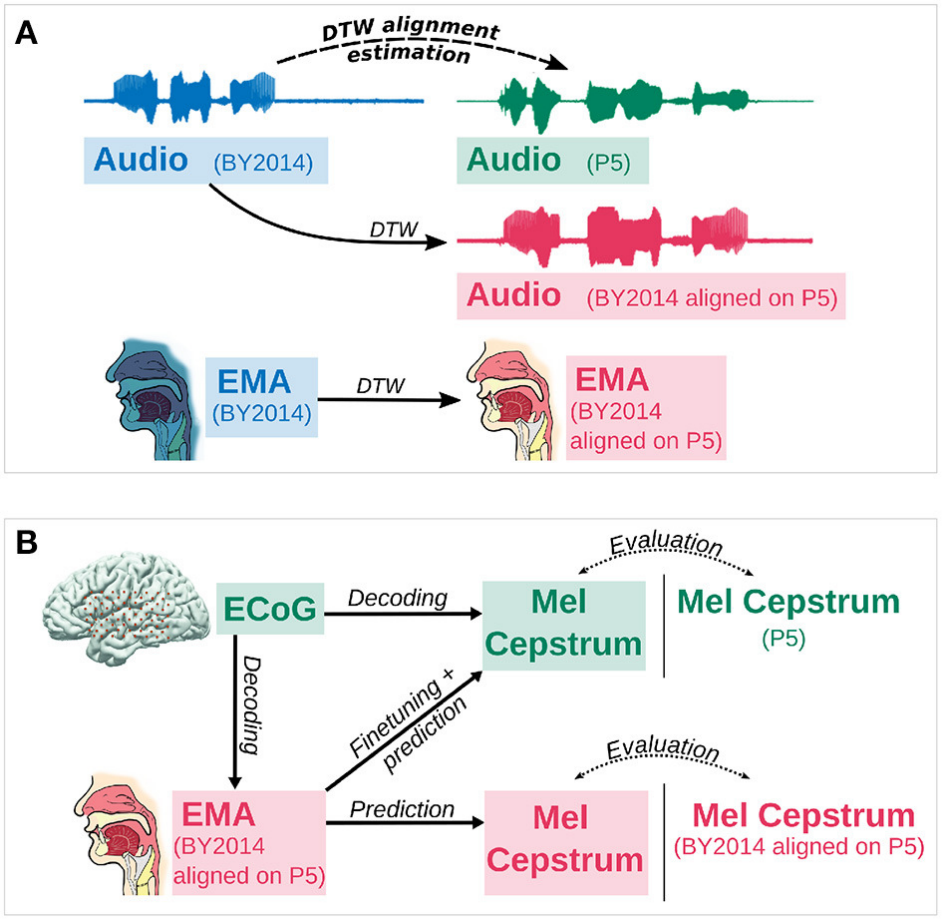
Evaluation of speech decoding from neural activity. **(A)** First, the optimal dynamic time warping alignment of BY2014 acoustic features onto P5 acoustic features is computed. Then this alignment is applied on articulatory trajectories of BY2014 to give an estimate of P5's articulatory trajectories. **(B)** Mel cepstral coefficients are decoded either 1. directly from neural activity or 2. from articulatory trajectories (EMA) decoded from neural activity. Mel cepstral coefficients directly decoded from neural activity or predicted from decoded articulatory trajectories with fine tuning are evaluated against the participant's original Mel cepstrum. Mel cepstral coefficients predicted from decoded articulatory trajectories without fine tuning are evaluated against BY2014's Mel cepstrum aligned on the participant's Mel cepstrum using DTW.

In order to decode articulatory trajectories from P5's neural activity, a dataset of synchronized ECoG recordings and articulatory trajectories of P5 were built from P5 recordings and BY2014. As articulatory trajectories of P5 were not recorded, they were estimated from those of the BY2014 corpus by aligning P5 and BY2014 audio recordings. Considering that the sentences recorded by P5 were part of BY2014 dataset, the optimal non-linear time distortion that maps an audio recording of BY2014 on to its corresponding P5 audio recording was computed using dynamic time warping (DTW; Sakoe and Chiba, 1978). This optimal transformation was then applied to the articulatory trajectories of BY2014 in order to estimate articulatory trajectories for P5 (Figure 1A). The estimated trajectories were therefore synchronized with P5's audio and ECoG recordings.

The Matlab DTW implementation we used required both signals to have the same number of samples, which was not the case as sentences of different speakers obviously have different durations. Signals were therefore resampled to have the same number of samples using Matlab's *interp1* function with “*pchip”* method (Piecewise Cubic Hermite Interpolating Polynomial) prior to running the DTW. In order to not introduce side effects that would influence the DTW, signals were padded by symmetrizing their sides before resampling. A simple euclidean distance was used as a sample-wise metric for the dynamic programming algorithm.

Even though P5 and BY2014 datasets contain the same sentences, they were spoken by different speakers of different genders. Moreover the EMA coils disturbed BY2014's speech. Thus, the tone, prosody, pitch and pronunciation of both speakers' recordings were different. To obtain an optimal alignment, the DTW was computed on the concatenated F0, Mel cepstral coefficients, and a boolean distinguishing speech and silent samples. Silence and speech were respectively labeled with 0 and 1 by an automatic speech detection algorithm based on audio envelope (see Section 2.2.2). F0 was set to 0 when no voicing was detected, which carries another boolean information about voicing. As DTW can be sensitive to misdetections of speech and F0 caused by background noise, both speech and voicing detections shorter than 50 ms where filtered out by setting the speech and F0 to 0. Formally, the resulting sample-wise distance between two sentences *S*_1_, *S*_2_ can thus be written as:


$$\begin{array}{l} d\left(S_{1}\left(t\right),S_{2}\left(t\right)\right)=\overset{24}{\underset{m=0}{\sum }}{\left({c_{1}}_{m}\left(t\right)-{c_{2}}_{m}\left(t\right)\right)}^{2}\\ \;+{\left(F0_{1}\left(t\right)-F0_{2}\left(t\right)\right)}^{2}\\ \;+{\left(speech_{1}\left(t\right)-speech_{2}\left(t\right)\right)}^{2} \end{array}$$

Where [(*c*_1_*m*__)_0≤*m*≤24_, (*c*_2_*m*__)_0≤*m*≤24_] are the Mel cepstral coefficients of [*S*_1_, *S*_2_] normalized by the absolute maximum value of the first Mel coefficient (representing the power of the signal), and [*F*0_1_, *F*0_2_] are the F0 of [*S*_1_, *S*_2_] normalized by their maximum value. The normalization of the features ensured that no feature overly contributes to the Euclidean distance and therefore to the alignment. Finally, a grid search was performed to test multiple weighting of each features using Pearson correlations for evaluation, showing best performance when using equal weights for all 3 features.

### 2.5. Articulatory synthesis

In order to reconstruct speech from decoded articulatory trajectories, a real-time-compatible articulatory-to-speech synthesizer was designed to predict Mel features from articulatory features.

A feedforward Deep Neural Network (DNN) was trained on BY2014 to predict Mel cepstral coefficients from articulatory trajectories. The DNN consisted of 3 hidden layers of 512 neurons each with *tanh* activation and a mean squared error loss function, parameters which were selected after a grid search. A sample ${\hat{\text{y}}}_{s}$ of Mel cepstrum was predicted from 10 past samples and 1 future sample of articulatory trajectories ${\left[{\text{x}}_{s-10}^{⊺},\ldots ,{\text{x}}_{s}^{⊺},{\text{x}}_{s+1}^{⊺}\right]}^{⊺}$. This time context was chosen after preliminary experiments including both objective and perceptive evaluations. Previous work already showed that adding a past time context to the network input was key to improve overall performance (Bocquelet et al., 2016c). The future time context further improved the overall decoding while adding a latency of 10 ms, which should not cause issues for a real-time use (Lee, 1950; Stuart et al., 2002).

Training was performed on a random split of 80% of BY2014, leaving 10% for validation and 10% for evaluation of the grid search parameters. The DNN was trained using Adam optimizer, with 25% dropout and batches of 32. In order to prevent overfitting, training was automatically stopped using early stopping with a patience of 20 epochs.

### 2.6. Neural decoding of speech

A source-filter representation of speech based on Mel cepstrum and F0 was decoded from ECoG features by regression methods. Two different paradigms were investigated to decode Mel cepstral coefficients: (1) direct decoding of Mel cepstral coefficients using linear methods, and (2) decoding of articulatory trajectories using linear methods, followed by an articulatory-to-acoustic neural network transforming these articulatory trajectories into the corresponding Mel cepstral coefficients. In order to provide a source signal for speech synthesis, the F0 was directly decoded in both cases from ECoG features using linear methods.

#### 2.6.1. Reduction of neural features

The number of neural features extracted from ECoG recordings for a single time frame was very large (1,512). In order to train a linear regression over neural data, the number of neural features was thus further reduced by PCA or PLS (Partial Least Squares). PCA was computed before concatenation of time context, whereas PLS was computed after concatenation of time context.

#### 2.6.2. Linear decoders

Linear regression methods were trained to predict speech features from neural features. We evaluated a simple linear regression, as well as ridge regressions and a Partial Least Squares (PLS) regression. The 3 ridge regressions that were trained each computed their regularization parameter in a different fashion: (1) with the L-curve method, (2) with a cross-validation, and (3) also with a cross-validation but with a different regularization parameter for each output feature.

#### 2.6.3. Decoding paradigms

Two decoding paradigms were compared: (1) a direct decoding of acoustic features of speech from neural features and (2) an indirect decoding of acoustic features of speech from neural features through and articulatory representation (see Figure 1B).

##### 2.6.3.1. Direct decoding

Mel cepstral coefficients and F0 were directly predicted from neural features by training multiple linear methods described in Section 2.6.2. Prior to training regressions, input and target features were preprocessed regardless of their the neural or acoustic nature.

First, input and target features were z-scored using the mean and standard deviation computed on the training set. Second, a time delay was optionally applied between input and target data. Third, a PCA decomposition was optionally performed to reduce the number of neural features. Finally, various amounts of temporal context were added to neural features by concatenating past and future frames of neural activity to predict a single frame of acoustic features, as described in Section 2.2.5.

Linear regressions, with or without regularization were optionally combined with a PCA decomposition as described in the previous paragraph, while PLS by design performed feature reduction altogether with regression after any other preprocessing.

##### 2.6.3.2. Indirect decoding

Using the same linear decoding methods, articulatory features of speech were also decoded from neural activity. Mel cepstral coefficients were then predicted from decoded articulatory trajectories by an articulatory-to-acoustic neural network trained on BY2014, as described in Section 2.5.

The articulatory-to-acoustic neural networks were trained to predict BY2014's Mel cepstral coefficients from BY2014's articulatory trajectories. Although P5 dataset's articulatory trajectories were estimated from BY2014, their temporal structure was different. Neural models were therefore **fine-tuned** to better fit the participant's data: the network's weights after training on BY2014 were used as initialization weights for training the model to predict the participant's Mel cepstral coefficients from its decoded articulatory trajectories. With the exception of the neural network initialization using a pretrained model, the training method is exactly the same as the one described in Section 2.5. With fine tuning, the articulatory-to-acoustic neural model predicted the participant's Mel cepstrum instead of BY2014's.

#### 2.6.4. Evaluation framework

Decoding methods were evaluated by comparing the speech features predicted from brain activity with the true features. The decoding models were evaluated on all the data using a 10-fold cross-validation.

##### 2.6.4.1. Cross-validation

Every decoding method (linear and DNN) were evaluated on a **10-fold cross-validation**. The set of 641 sentences was randomly split in 10 folds that contained approximately the same number of sentences. One fold constituted the testing set, the others constituted the training set. Each fold was used for testing once until the models were evaluated on all the corpus.

In the case of the indirect decoding paradigm, the articulatory-to-acoustic DNN was fine-tuned inside the cross-validation on the same training set and was evaluated on the test set. For that, it required a complete dataset of decoded articulatory trajectories, including training and testing sets. Therefore articulatory trajectories were decoded on all 10 folds by the linear decoder that was trained on the 9 training folds.

Mean and standard deviation of the training set were computed in each fold and were used to z-score both the training set and the testing set. PCA was also computed on the training set and then applied to both training and testing sets in each fold.

##### 2.6.4.2. Evaluation of predicted speech

Decoding methods were evaluated by comparing predicted features with ground truth features using Pearson correlation and mean squared error computed over entire sentences, leading to one value per sentence.

Features predicted from direct decoding methods were compared to the participant's ground truth features. Mel cepstral coefficients predicted from articulatory methods with fine tuning were also compared with the participant's Mel cepstral coefficients, while Mel features predicted without fine tuning were compared to BY2014's Mel cepstral coefficients aligned on the participant's features by DTW (Figure 1B).

Chance levels were estimated by randomly shuffling neural data samples and running the complete decoding and evaluation pipeline in the exact same way.

##### 2.6.4.3. Statistical evaluation

Statistical significance between Pearson correlations of decoded features and their corresponding chance levels was computed using a Bonferroni-corrected Wilcoxon signed-rank test. Pair-wise statistical significance between Pearson correlations of decoded features using different decoding conditions was computed using a Quade-Conover test.

## 3. Results

The results presented here are all displayed using violin plots, where each dot of the violin plots shows the Pearson correlations of a single sentence for a given decoded feature with the corresponding ground truth.

### 3.1. Comparing linear methods for direct speech decoding

**Figure 2:**
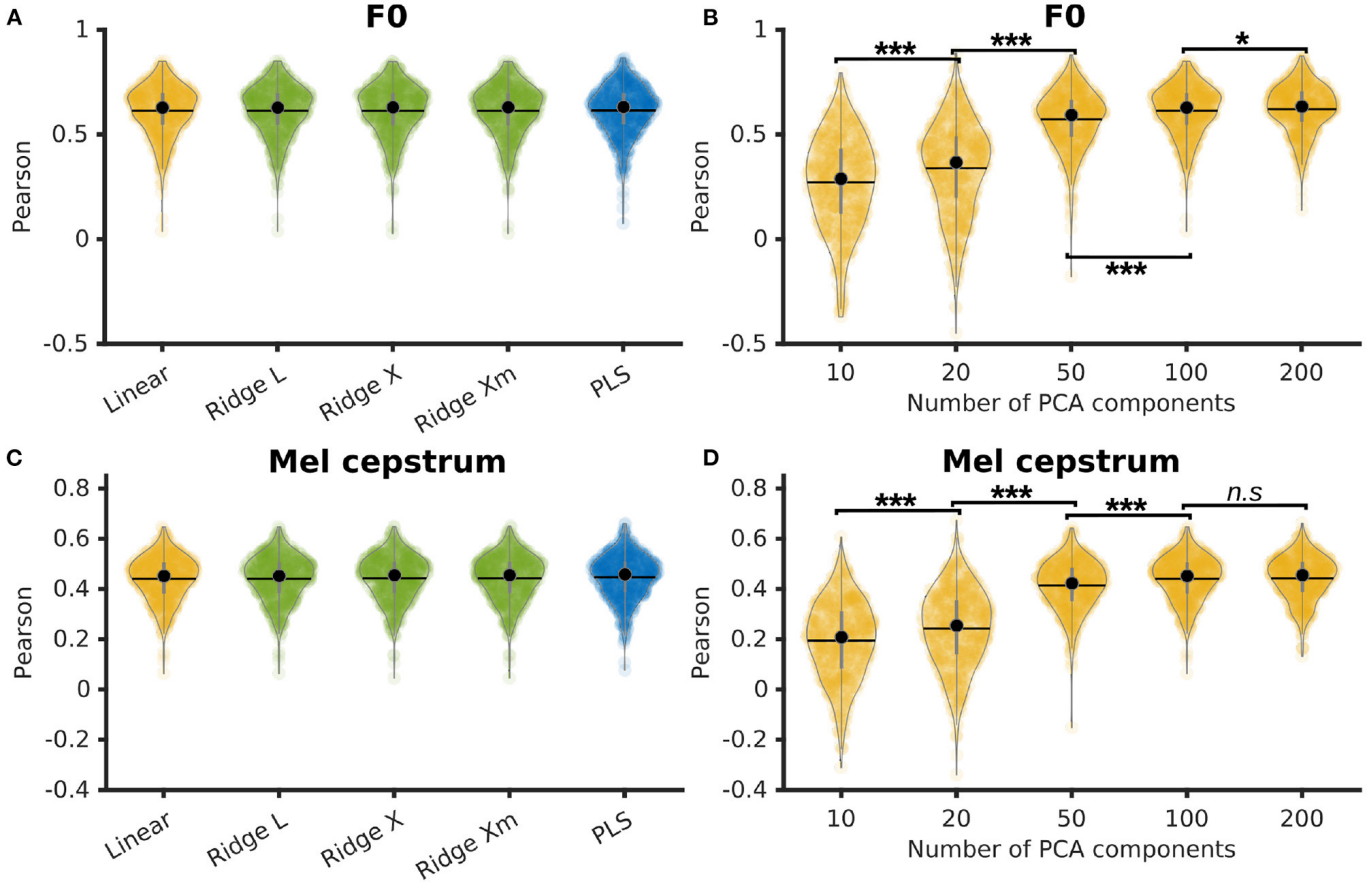
Comparison of several linear methods for direct decoding of acoustic features of speech. **(A)** Pearson correlations of decoded F0 from linear methods. Ridge and linear regressions were trained on the first 100 PCA components of neural activity and PLS regression was trained with 12 components. Ridge regressions were trained using 3 different methods to compute the λ factor: L-curve (L), cross-validation (X), and cross-validation with individual λ per features (Xm). **(B)** Pearson correlations of decoded F0 using a linear regression trained on varying PCA reductions of neural activity. Statistical significance computed by Quade-Conover test [Quade test: *p* < 0.001, *t*_(4,2516)_ = 834.8]. **(C)** Pearson correlations of decoded Mel cepstrum from linear methods. Ridge and linear regressions were trained on the first 100 PCA components of neural activity and PLS regression was trained with 12 components. Ridge regressions were trained using 3 different methods to compute the λ factor: L-curve (L), cross-validation (X) and cross-validation with individual λ per features (Xm). **(D)** Pearson correlations of decoded Mel cepstrum using a linear regression trained on varying PCA reductions of neural activity. Statistical significance computed by Quade-Conover test [Quade test: *p* < 0.001, *t*_(4,2516)_ = 837.4]. Conover comparisons significance for **(B, D)**: *n.s*: *p* ≥ 0.05 [**(D)**: *t*_(2516)_ = 0.5], *p = 0.013 [**(B)**: *t*_(2516)_ = 2.5], ***p < 0.001 [**(B, D)**: *t*_(2516)_ > 6.5].

P5's F0 and Mel cesptrum were decoded using a simple linear regression, a PLS regression, and ridge regressions with 3 different ways to compute the λ factor: L-curve, cross-validation, and cross-validation with individual λ per feature. The linear and ridge regressions were trained on a PCA reduction of the neural features down to 100 features with 0 ms of time delay and 210 ms of time context (10 frames of past context and 10 frames of future context). The PLS regression was trained using 12 components and the same time delay and context. Pearson correlations of decoded F0 achieved similar results for all methods with median correlations of 0.63 ± 0.12 for all regressions (Figure 2A). Pearson correlations of decoded Mel cepstrum achieved similar results for all methods with median correlations of 0.45 ± 0.09 for linear regression and 0.46 ± 0.09 for ridge and PLS regressions (Figure 2C).

The influence of the PCA reduction on linear decoding of P5's F0 and Mel cepstrum was evaluated by training linear regressions with 0 ms of delay and 210 ms of time context on 10, 20, 50, 100, and 200 PCA components of P5's neural features. Best median Pearson correlations of decoded F0 (Figure 2B) was found for 200 PCA components (0.63 ± 0.11), which was found to be significantly higher than smaller numbers of components. Best median Pearson correlations of decoded Mel cepstrum (Figure 2D) was found for 100 PCA components (0.45 ± 0.09) and 200 PCA components (0.46 ± 0.09), which were found to be significantly higher than smaller numbers of components.

### 3.2. Direct speech decoding using PLS regression

**Figure 3:**
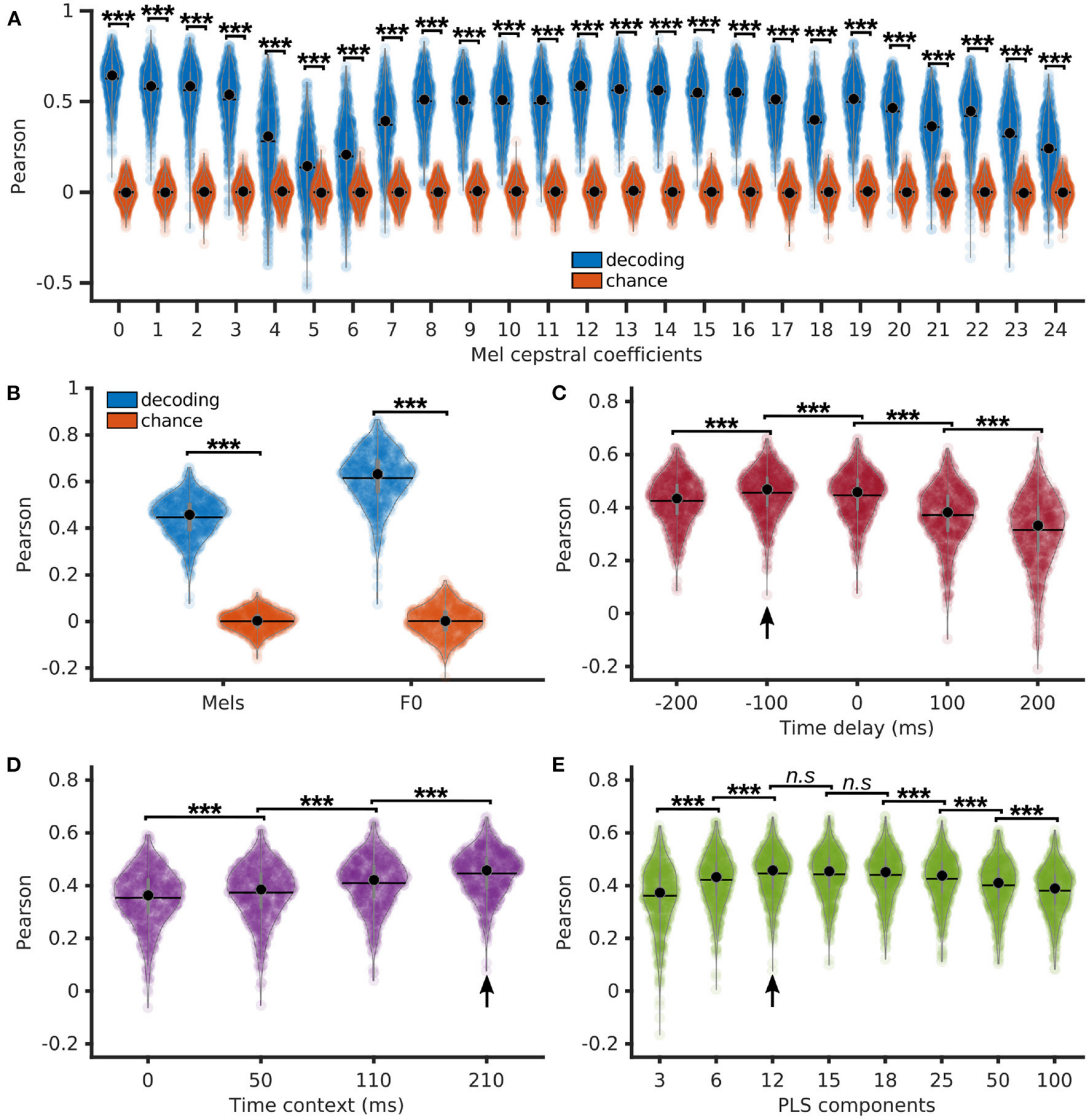
Direct decoding of acoustic features of speech using PLS regression. **(A)** Pearson correlations of decoded Mel cepstral coefficients (blue) and the corresponding chance levels (red) using a PLS regression with 12 components, 210 ms of time context and 0 ms of time delay. **(B)** Average Pearson correlations of decoded Mel cepstrum across coefficients and Pearson correlations of decoded F0 using a PLS regression with 12 components, 210 ms of time context and 0 ms of time delay. **(C)** Pearson correlations of decoded Mel cepstrum with varying time delay using PLS regression with 12 components and 210 ms of time context. **(D)** Pearson correlations of decoded Mel cepstrum with varying time context using PLS regression with 12 components and 0 ms of time delay. **(E)** Pearson correlations of decoded Mel cepstrum using PLS regression with varying number of components, 210 ms of time context and 0 ms of time delay. Statistical significance with respect to chance levels computed with Bonferroni-corrected Wilcoxon signed rank test for **(A, B)** (see values in Section 3.2). Statistical significance computed by Quade-Conover test for **(C)** [Quade test: *p* < 0.001, *t*_(4,2516)_ = 399.8], **(D)** [Quade test: *p* < 0.001, *t*_(3,1887)_ = 1388.7] and **(E)** [Quade test: *p* < 0.001, *t*_(7,4403)_ = 254.9]. Conover comparisons for **(C–E)**: *n.s*: *p* ≥ 0.05 [**(E)**: *t*_(4403)_ < 1.7], ***p < 0.001 [**(C)**: *t*_(2516)_ > 4.6, **(D)**: *t*_(1887)_ > 18.8, **(E)**: *t*_(4403)_>7.3]. Arrows indicate best accuracies.

We next detail the reconstruction accuracy of individual Mel cepstral coefficients and F0 obtained with a PLS regression with 12 components, 210 ms of context, and no delay. Pearson correlations of decoded Mel cepstral coefficients with their corresponding ground truth were computed on each sentence, showing to be significantly higher than chance (*p* < < 0.001, *z* ∈ [15.0, 17.6], for each Mel cepstral coefficient) using a Bonferroni-corrected Wilcoxon signed-rank test (Figure 3A). Pearson correlations of the decoded F0 and average Pearson correlations of the decoded Mel cepstral coefficients were also significantly higher than chance (Figure 3B).

We further evaluated the influence of delay and context on decoding accuracy. Time delays of −200, −100, 0, 100, and 200 ms were introduced between neural features and Mel cepstrum prior to decoding with a PLS regression with 12 components and 210 ms of time context (Figure 3C). Best decoding was found for −100 ms of delay (*median* = 0.47 ± 0.09), which corresponded to decoding a frame of acoustic speech using neural features over the last 200 ms. Time contexts of 0, 50, 110, and 210 ms were evaluated for decoding of Mel cepstrum using a PLS regression with 12 components and no delay (Figure 3D). The best median Pearson correlation was found for the largest 210 ms context (0.46 ± 0.09), which was found to be significantly higher than smaller contexts.

Finally, PLS regressions with 3, 6, 12, 15, 18, 25, 50, and 100 components were compared for Mel cepstrum decoding from neural features. All regressions used 0 ms of delay and 210 ms of time context. Best median Pearson correlation was found for 12 components (0.46 ± 0.09), which was found to perform significantly better decoding compared to 3, 6, 25, and 50 components (Figure 3E). However no statistical differences were found between 12, 15, and 18 components.

### 3.3. Indirect speech decoding using PLS regression

**Figure 4:**
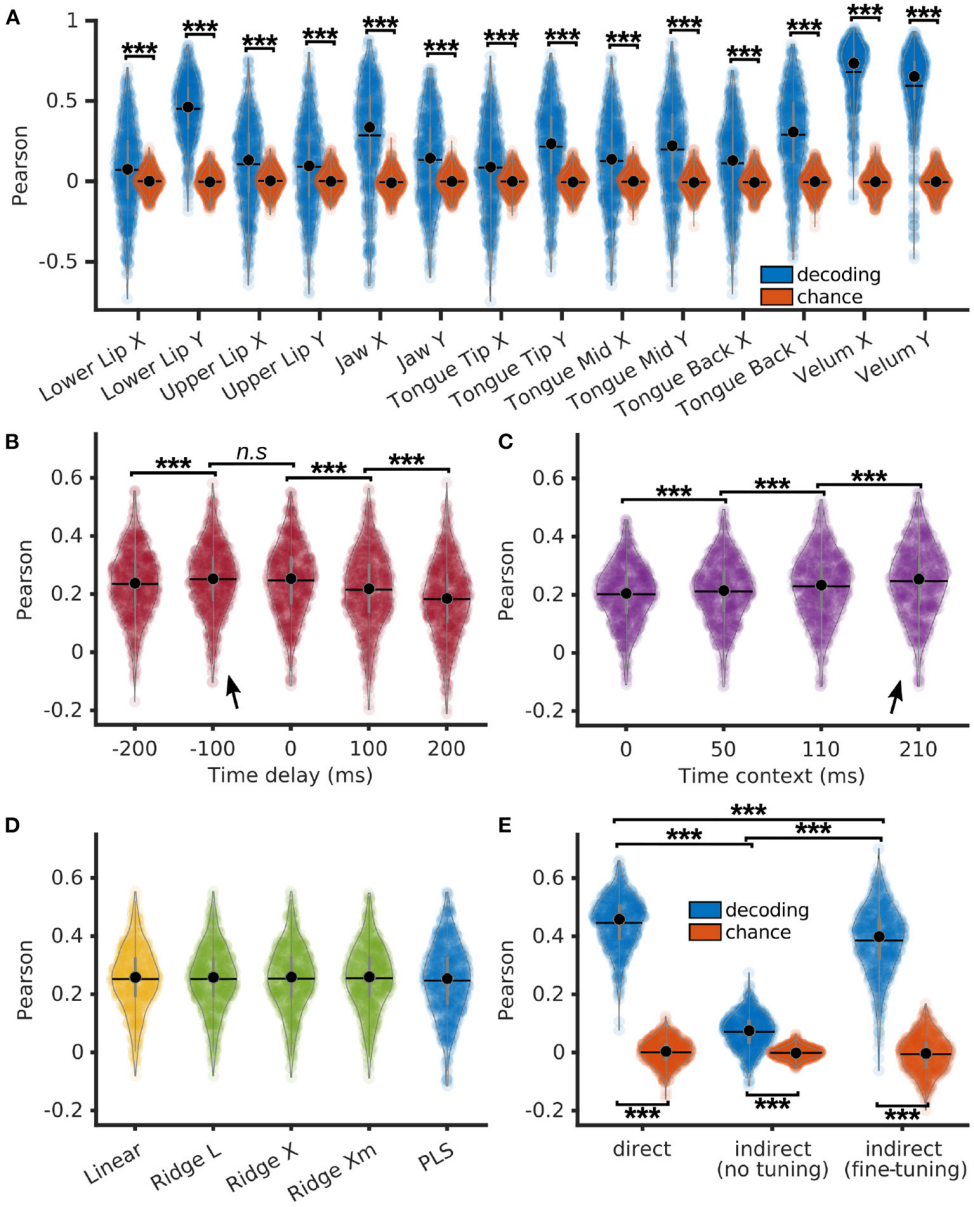
Indirect decoding of Mel cepstrum from brain activity through an articulatory representation using PLS regression. **(A)** Pearson correlations of decoded EMA (blue) and their matching chance levels (red) using PLS regression with 12 components. **(B)** Pearson correlations of decoded EMA using PLS regression with 12 components, 210 ms of time context and varying time delays. **(C)** Pearson correlations of decoded EMA using PLS regression with 12 components, 0 ms of delay and varying time contexts. **(D)** Pearson correlations of decoded EMA using linear/ridge regressions with PCA reduction (100 components) and PLS regressions with 12 components. Ridge regressions were trained using 3 different methods to compute the λ factor: L-curve (L), cross-validation (X) and cross-validation with individual λ per features (Xm). **(E)** Pearson correlations of decoded Mel cepstrum (blue) and their matching chance levels (red) using either 1. direct decoding with PLS regression (direct), 2. indirect prediction from decoded EMA with a articulatory-to-acoustic DNN without fine tuning or 3. indirect prediction with fine tuning. Statistical significance with respect to chance levels on **(A, E)** computed with Bonferroni-corrected Wilcoxon signed-rank test (see values in Section 3.3). Statistical significance computed by Quade-Conover test for **(B)** [Quade test: *p* < 0.001, *t*_(4,2516)_ = 75.1], **(C)** [Quade test: *p* < 0.001, *t*_(3,1887)_ = 369.2], and **(E)** [Quade test: *p* < 0.001, *t*_(2,1258)_ = 1033.1]. Conover comparisons for **(B–E)**: *n.s*: *p* ≥ 0.05 [**(B)**: *t*_(2516)_ = 0.99], ***p < 0.001 [**(B)**: *t*_(2516)_ > 4, 8, **(C)**: *t*_(1887)_ > 8.8, **(E)**: *t*_(1258)_ > 29.9]. Arrows indicate best accuracies.

Articulatory features of each P5 sentences were decoded from neural features using a PLS regression with 12 components, 210 ms of context (10 frames of past context and 10 frames of future context), and no delay. Pearson correlations of decoded articulatory features with their corresponding ground truth were computed on each sentence, showing to be significantly higher than chance (*p* < 0.001, *z* ∈ [6.5, 21.7], for each articulatory features) using a Bonferroni-corrected Wilcoxon signed rank test (Figure 4A).

Time delays of −200, −100, 0, 100, and 200 ms were introduced between neural and articulatory features prior to decoding with a PLS regression with 12 components and 210 ms of time context (Figure 4B). Average Pearson correlations of decoded sentences across articulatory features were found to be significantly higher for −100 and 0 ms delays. Best overall median correlation was found for −100 ms of delay (0.25 ± 0.11), which corresponded to decoding a frame of acoustic speech using the last 200 to 0 ms of neural features.

Time contexts of 0, 50, 110, and 210 ms were evaluated for decoding of articulatory features using a PLS regression with 12 components and no context (Figure 4C). The best median Pearson correlation was found for the largest 210 ms context (0.25 ± 0.12), which was significantly higher than smaller contexts.

The decoding accuracy of articulatory features was compared across the 5 linear methods with 0 ms of time delay and 210 ms of time context: a simple linear regression, ridge regressions with 3 different ways to compute the λ factor: L-curve, cross-validation, and cross-validation with individual λ per feature, and finally a PLS regression with 12 components. Linear and ridge regressions were trained on a PCA reduction of the neural features down to 100 features. Pearson correlations of decoded articulatory features achieved similar results for all methods with median correlations up to 0.26 ± 0.11 for the ridge regressions with cross-validation, 0.26 ± 0.11 for the linear regression and 0.25 ± 0.12 for the PLS regression (Figure 4D).

Finally, direct and indirect decoding of P5's Mel cepstrum were compared. A PLS regression with 12 components, 0 ms of time delay and 210 ms of time context was trained to decode Mel cepstrum and articulatory features from P5's neural features. The articulatory-to-acoustic DNN trained on BY2014 (see Section 2.5) predicted the Mel cepstrum from decoded articulatory trajectories. Both indirect decoding of Mel cepstrum with and without fine tuning of the DNN were compared. Mel cepstral coefficients were decoded well above chance level with both direct and indirect methods (Figure 4E, Bonferroni-corrected signed rank test: *p* < < 0.001; *z* = {21.7, 19.6, 21.7} for direct decoding, indirect decoding without finetuning, and indirect decoding with finetuning, respectively). Pearson correlations of directly and indirectly decoded Mel cepstrums were all found to be statistically different. Best Pearson correlations were achieved by direct decoding (*median* = 0.46 ± 0.09), followed by indirect decoding with fine tuning of the DNN (*median* = 0.40 ± 0.11), while worst correlations were achieved by indirect decoding of the DNN without fine tuning (*median* = 0.08 ± 0.06).

### 3.4. Influence of frontal and temporal electrodes on speech decoding

**Figure 5:**
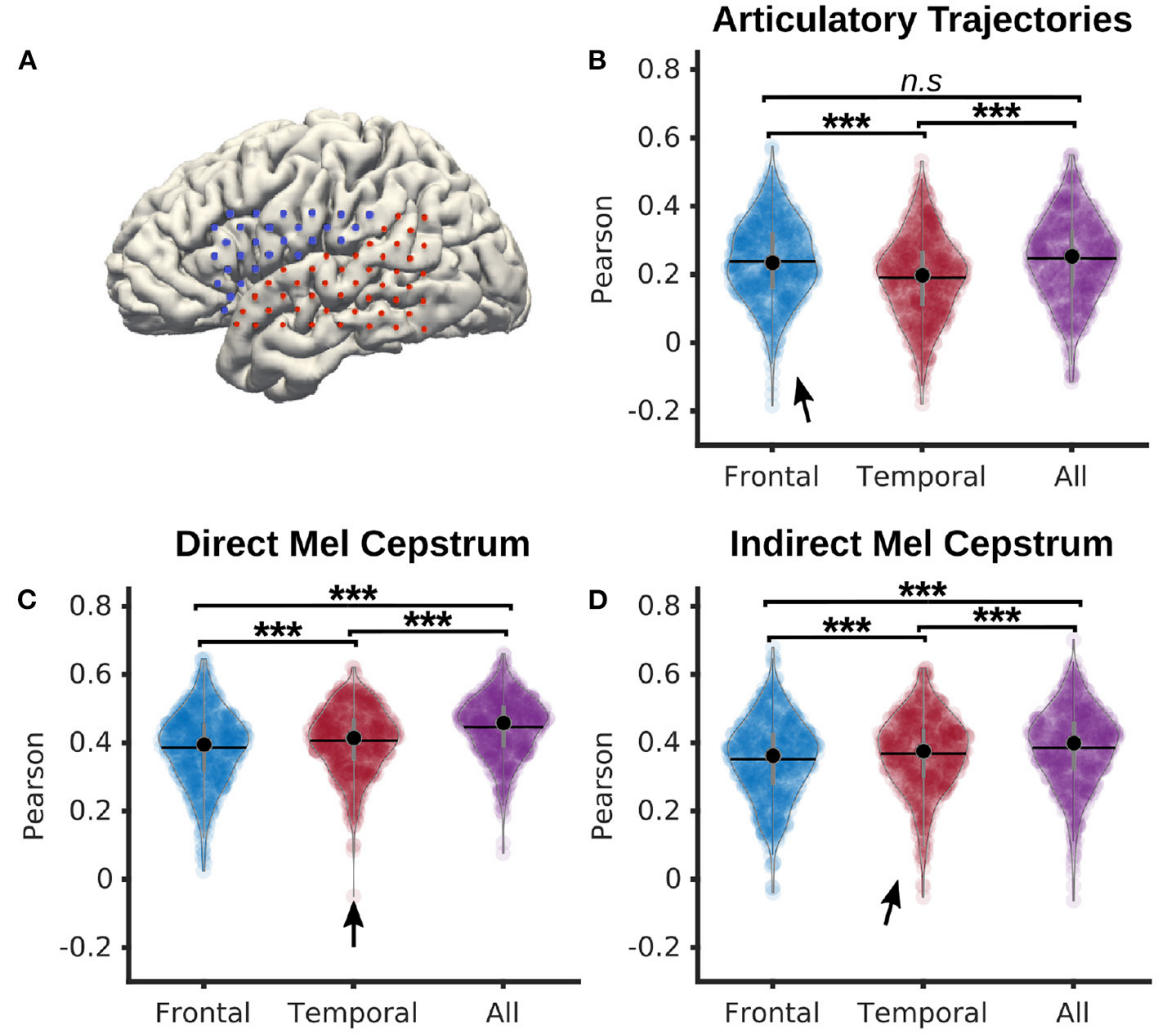
Comparison of frontal and temporal electrodes for decoding of speech from P5 dataset. **(A)** Map of frontal (blue) and temporal (red) electrodes of P5 dataset. **(B)** Pearson correlations of articulatory trajectories decoded by a PLS regression with 12 components from either frontal, temporal, or all electrodes (median correlations *r* = 0.24, *r* = 0.20, *r* = 0.25 for frontal, temporal, and all electrodes, respectively). **(C)** Pearson correlations of Mel cepstral coefficients decoded by a PLS regression with 12 components from either frontal, temporal, or all electrodes (median correlations *r* = 0.39, *r* = 0.40, *r* = 0.45 for frontal, temporal, and all electrodes, respectively). **(D)** Pearson correlations of Mel cepstral coefficients predicted from decoded articulatory trajectories using an articulatory-to-acoustic DNN from either frontal, temporal, or all electrodes (median correlations *r* = 0.38, *r* = 0.38, *r* = 0.41 for frontal, temporal, and all electrodes respectively). Statistical significance computed using Quade-Conover. Quade tests: *p* < 0.001 [*t*_(2,1258)_ > 30.5]. Conover comparisons for **(B–D)**: *n.s*: *p* ≥ 0.05 [**(B)**: *t*_(1258)_ = 0.8], ***p < 0.001 [**(B–D)**: *t*_(1258)_ > 3.6]. Arrows indicate best accuracies.

Decoding from frontal, temporal, and all electrodes (Figure 5A) was compared using a PLS regression with 12 components, 0 ms of delay and 210 ms of time context. Statistical significance was computed using a Quade-Conover test.

Pearson correlations of decoded articulatory trajectories (Figure 5B) from frontal electrodes (*median* = 0.24 ± 0.12) were significantly higher than from temporal electrodes (*median* = 0.20 ± 0.12). Highest correlations were reported using all electrodes (*median* = 0.25 ± 0.12), although not significantly higher than with frontal electrodes.

Pearson correlations of directly decoded Mel cepstrums (Figure 5C) from temporal electrodes (*median* = 0.41 ± 0.09) were significantly higher than from frontal electrodes (*median* = 0.40 ± 0.11). Decoding using all electrodes (*median* = 0.46 ± 0.09) achieved significantly higher correlations than using only frontal or temporal electrodes.

Pearson correlations of indirectly decoded Mel cepstrums (Figure 5D) from temporal electrodes using a fine-tuned DNN (*median* = 0.38 ± 0.11) were significantly higher than from frontal electrodes (*median* = 0.36 ± 0.12). Decoding correlations using all electrodes (*median* = 0.40 ± 0.11) achieved significantly higher correlations than using only frontal or temporal electrodes.

## 4. Discussion

We evaluated different linear methods for predicting speech from ECoG cortical activity. Our findings showed a similar performance for all linear models, vastly better than chance. In particular, PLS regression, which was previously used for motor BCIs (Chao et al., 2010; Eliseyev et al., 2012), was evaluated for the first time for speech decoding from cortical activity. Our findings are consistent and extend a previous study aiming at decoding spectrograms from ECoG activity (Martin et al., 2014). The more compact latent space used by the PLS regression makes it a good candidate for a speech BCI compared to a linear regression using PCA, as it should reduce overfitting and offer lower dimensional controls for the user.

In order to run the decoding models, the dimensionality of the neural data should first be reduced. We investigated both PCA and PLS reductions. Our results showed that PCA-based linear decoding of acoustic features of speech improved with the number of PCA components, with a maximum at 100 components for the Mel cepstrum and 200 components for the F0. We did not test more features, as the 256-GB memory of our computing server was maxed out. In these experiments, the PCA was computed before concatenating frames for time context, as a preliminary experiment showed that computing PCA after temporal context decreased decoding correlations. On the other hand, feature reduction using PLS showed best correlation for 12–18 components. This cannot be directly compared with the PCA results, as the PLS reduction was computed after concatenating frames for time context. However, in order to assess the best feature representation for decoding, the PLS reduction showed a much more compact representation for similar decoding performance, with 12 components appearing as a good number for designing a speech BCI. This result can be paralleled with previous work reporting intelligible speech reconstruction from 10 to 12 articulatory trajectories (Bocquelet et al., 2014).

We investigated the influence of the time context window of neural activity used for decoding acoustic and articulatory features of speech. Our findings showed that increasing the size of this window improved decoding, up to 210 ms. While further increasing the time window might still improve decoding, we could not test it on the whole dataset as it was maxing out the RAM of our computing server. However, on prior experiments on a smaller subset of our dataset, we found that increasing time context up to 310 ms actually decreased decoding correlations compared to 210 ms context. By evaluating the optimal time delay between neural activity and speech, we found that best decoding was achieved by using neural activity from the past 210 ms. This result tends to show that speech was actually decoded from neural activity related to speech intent more than auditory and sensory feedback. On a practical side, decoding speech from that optimal time window would be real-time compatible for a closed-loop speech BCI.

Cortical activity related to speech articulators is mainly found in frontal areas, while activity related to acoustic processing is predominantly found in temporal areas. Although the differences were small, we found that decoding acoustic features of speech from neural activity performed significantly better using temporal electrodes than frontal electrodes, and that decoding articulatory trajectories from neural activity performed significantly better using frontal electrodes than temporal electrodes. For both acoustic and articulatory speech features however, using all electrodes for speech decoding performed significantly better than using only frontal or temporal electrodes. Therefore, frontal and temporal electrodes contain at least some non-overlapping information about the representation of produced speech, which further supports the current understanding of cortical mechanisms of speech as distributed cortical processes across frontal and temporal regions (Hickok and Poeppel, 2007; Tourville and Guenther, 2011). A speech BCI might benefit from considering cortical signals distributed over multiple areas.

Finally, we compared two decoding paradigms: (1) direct decoding of acoustic features of speech using linear methods, and (2) indirect decoding of acoustic features of speech by first decoding articulatory trajectories from cortical activity using linear methods and feeding them to a DNN-based articulatory-to-acoustic synthesizer. We found that fine tuning the pretrained articulatory-to-acoustic DNN on the participant data was essential to get a good performance of indirect decoding. Yet, direct decoding performed better than indirect decoding in opposition with previous work using neural networks (Anumanchipalli et al., 2019). This discrepancy could possibly be due to a difference in the quality of the reconstruction of articulatory trajectories from ECoG, with a superiority of DNN-based decoder (achieving correlations around 0.65) as compared to the linear decoders used in the present study (achieving correlations around 0.25 as shown in Figure 4D). However such difference was not observed for direct Mel cepstrum reconstruction (with correlations of 0.55 with DNNs and 0.45 with linear methods) as supported by recent near real-time speech decoding studies (Anumanchipalli et al., 2019; Makin et al., 2020; Moses et al., 2021). Another possible reason for the better direct decoding could be a suboptimal estimation of the articulatory trajectories using DTW. While we checked that DTW provided coherent resynthesis after alignment of BY2014 with P5 corpus, an acoustic-to-articulatory inversion method using DNN trained on multiple datasets could be more robust for indirect speech reconstruction (Anumanchipalli et al., 2019).

The direct and indirect decoding methods used in this study as well as the data processing are all compatible with real-time use for a natural speech BCI, including speech synthesis from the Mel cepstral coefficients and F0, which would allow continuous and arbitrary speech reconstruction from speech-related cortical activity with a minimal latency. However, we did not achieve intelligibility using linear methods (two examples are provided in Supplementary material: audio 1 contains a set of decoded French vowels “/a/ /i/ /u/” and audio 2 contains the decoded French sentence “*C'est désormais chose faite*”). We believe that real-time compatible neural networks may improve speech decoding from cortical activity. In order to remain compatible with a natural speech BCI, those would have to be designed to predict acoustic features of speech frame by frame with a millisecond-scale latency.

## Data availability statement

The datasets presented in this article are not readily available because the speech data associated to the ECoG data used in this study may reveal the identity of the patient. The raw data will thus only be made available under specific agreement with the Grenoble University Hospital upon reasonable request to the corresponding author. Requests to access the datasets should be directed to blaise.yvert@inserm.fr.

## Ethics statement

The studies involving human participants were reviewed and approved by the French regulatory agency ANSM (DMDPT-TECH/MM/2015-A00108-41) and the Comité de Protection des Personnes Sud Est V (CPP-15-CHUG-12). The patients/participants provided their written informed consent to participate in this study.

## Author contributions

All analysis was performed by GL and PR under the supervision of BY. Data preprocessing was performed by FB, PR, and GL. Acoustic contamination analysis was performed by PR. GL processed the data and implemented and evaluated the different decoders. PR, FB, MA, PK, SC, and BY participated to the recording of P5 dataset. GL and BY wrote the manuscript. BY designed and coordinated this study. All authors contributed to the article and approved the submitted version.
