# Supplementary material for: Overt speech decoding from cortical activity: a comparison of different linear methods
Source: Front Hum Neurosci. 2023 Jun 23;17:1124065. doi: 10.3389/fnhum.2023.1124065 (PMC10326283; doi:10.3389/fnhum.2023.1124065)
Supplement: Supplementary file 1 [file Data_Sheet_1.PDF]

Supplementary Material

1 Supplementary Data

We provide two examples of sentences decoded from P5 neural activity using a PLS regression with 12 components, 210ms of time context and 0ms of time delay. Although the correlations of the decoded sentences with ground truth sentences were significantly higher than chance, the decoded sentences did not reach intelligibility.

audio 1: Sequence of three french vowels "/a/ /i/ /u/"

audio 2: French sentence "*C'est désormais chose faite*"
